# Minimal role of interleukin 6 and toll-like receptor 2 and 4 in murine models of immune-mediated bone marrow failure

**DOI:** 10.1371/journal.pone.0248343

**Published:** 2021-03-12

**Authors:** Sabrina Solorzano, Jisoo Kim, Jichun Chen, Xingmin Feng, Neal S. Young

**Affiliations:** 1 Hematology Branch, National Heart, Lung, and Blood Institute, National Institutes of Health, Bethesda, Maryland, United States of America; 2 Center for Cancer and Blood Disorders, Children’s National Medical Center, Washington DC, United States of America; University of Kentucky, UNITED STATES

## Abstract

Immune aplastic anemia (AA) results from T cell attack on hematopoietic cells, resulting in bone marrow hypocellularity and pancytopenia. Animal models have been successfully developed to study pathophysiological mechanisms in AA. While we have systemically defined the critical components of the adaptive immune response in the pathogenesis of immune marrow failure using this model, the role of innate immunity has not been fully investigated. Here, we demonstrate that lymph node (LN) cells from B6-based donor mice carrying IL-6, TLR2, or TLR4 gene deletions were fully functional in inducing severe pancytopenia and bone marrow failure (BMF) when infused into MHC-mismatched CByB6F1 recipients. Conversely, B6-based recipient mice with IL-6, TLR2, and TLR4 deletion backgrounds were all susceptible to immune-mediated BMF relative to wild-type B6 recipients following infusion of MHC-mismatched LN cells from FVB donors, but the disease appeared more severe in IL-6 deficient mice. We conclude that IL-6, TLR2, and TLR4, molecular elements important in maintenance of normal innate immunity, have limited roles in a murine model of immune-mediated BMF. Rather, adaptive immunity appears to be the major contributor to the animal disease.

## Background

Aplastic anemia (AA) is a life threatening form of bone marrow failure (BMF) involving pancytopenia in association with bone marrow (BM) hypoplasia or aplasia. Almost all sporadic AA, especially when severe and acute, appears to be immune mediated. Many studies have indicated the involvement of the immune system in AA, focusing on the role and activity of cytotoxic T cells [1]: these cells appear to be activated [2], produce type 1 cytokines [3, 4], and induce apoptosis via Fas/FasL [5]. Treg cells are decreased in patients with AA and increased with recovering hematologic response [6, 7]. The cascade of triggering antigens for this dominant T cell response remains unknown. Our laboratory has developed immune–mediated murine models and systematically defined the critical roles of acquired immunity components, especially CD4^+^ and CD8^+^ T cells as immune effectors [8, 9] orchestrating the destruction of target BM hematopoietic stem and progenitor cells (HSPCs) through a bystander process [10] mediated by interferon gamma (IFN-γ) [11] and tumor necrosis factor alpha (TNF-α) [12]. Both Th1 [13] and Th17 [14] immune responses were involved in disease progression leading to BM cell destruction through the Fas/FasL cell death pathway [15]. We also found limited involvement of the perforin and granzyme pathway [16] and a dispensable role of IL-18 [17] in BMF development. Macrophages have proved an active role in augmenting an immune response leading to BMF [12, 18]; still, the involvement of innate immunity has been less investigated. These results prompted us to pursue the potential roles of important components of innate immunity, such as toll-like receptors (TLRs) and inflammatory cytokine interleukin 6 (IL-6), to gain a broader picture of the molecular pathophysiology of the induced animal model and devastating human disease.

Toll-like receptors (TLRs) are pattern recognition receptors, critical in recognizing pathogen-associated molecular patterns and in initiating an immune response in the host. There are ten known TLRs in humans, expressed in cells of hematopoietic origin (e.g. dendritic cells, macrophages, lymphocytes, and hematopoietic stem and progenitor cells) and non-hematopoietic origin (e.g. endothelial and mesenchymal cells). Most TLRs are resident on the cell surface to interact with pathogenic ligands, while some are intracellular in endosomes to interact with pathogenic nucleic acids. TLRs also respond to damage-associated molecular patterns (DAMPs) that are endogenous ligands released at the site of tissue damage. TLRs play an important role in innate immunity, as they recognize various microbial components, for example lipopolysaccharide (LPS) found in bacteria cell walls or viral single-stranded RNA [19]. They have also been implicated in the pathogenesis of various autoimmune diseases such as rheumatoid arthritis (RA), systemic lupus erythematosus (SLE), and systemic sclerosis (SSc) [20]. The synovial tissues of patients with RA express elevated levels of TLRs, including TLR2 and TLR4 [21]. In murine models, protection from RA has been observed in mice with a TLR4 gene deletion [22]. Moreover, DAMPs, functioning as ligands for TLR4, have been found to be elevated in SSc skin and lung tissue [23]. The evidence of TLR involvement, specifically TLR2 and TLR4, in the pathogenesis of these autoimmune conditions suggested their possible involvement in the pathogenesis of immune AA.

The cytokine IL-6 has pleiotropic effects, including promotion of naïve CD4^+^ T cell differentiation, cytotoxic CD8^+^ T cell differentiation, and down regulation of Treg differentiation [24]. Murine models have demonstrated that IL-6 deficient mice fail to efficiently respond to various viruses, bacteria, and tissue damage [25]. In relation to TLR, murine models have demonstrated that deletion of TLR2 and TLR3 in mice halted IL-6 production [26, 27]. Additionally, IL-6 plays a pathogenic role in autoimmunity in numerous mouse models, and in human disease. IL-6 blockade in mice reduced susceptibility to RA [28, 29], SLE [30], SSc [31], experimental autoimmune uveoretinitis [32], and experimental autoimmune encephalomyelitis [33]. An anti-IL-6R monoclonal antibody is approved for and widely used in the treatment of RA [24, 34]. This evidence suggested a potential IL-6 involvement in the pathogenesis of immune AA.

Murine models have been instrumental in studies aimed at defining the roles of immune components in the development of BMF, but the importance of innate immunity cascades has not been previously investigated. Given the accumulating evidence of IL-6, TLR2 and TLR4 contributing to the pathogenesis of immune-mediated diseases, we exploited existing gene-deletion mouse models commercially available in the B6 genetic background to test the roles of IL-6, TLR2 and TLR4 in the development of BMF. Results from our study do not suggest a significant or direct role in IL-6, TLR2 or TLR4 in the murine model of immune BMF.

## Methods

### Animals and induction of BMF

Inbred C57BL/6 (B6) and FVB/NJ (FVB), hybrid BALB/cBy × B6 F1(CByB6F1), induced mutant B6.129S2-*Il6*^*tm1Kopf*^/J (IL-6^-/-^) and B6.129-*Tlr2*^*tm1Kir*^/J (TLR2^-/-^), and spontaneous mutant B6.B10ScN-*Tlr4*^*lps-del*^/JthJ (TLR4^-/-^) mice were originally obtained from the Jackson Laboratory (Bar Harbor, ME) and were bred and maintained in National Institutes of Health animal facilities under standard care and nutrition. The Animal Care and Use Committee at the National Heart, Lung, and Blood Institute approved all animal studies. In one model, inguinal, axillary, and lateral axillary lymph nodes (LN) were removed from B6, IL-6^-/-^, TLR2^-/-^, or TLR4^-/-^ donors, homogenized in RPMI 1640 media supplemented with fetal bovine serum, penicillium, streptomycin and glutamine using a mini-tissue grinder (A. Daigger & Company, Vernon Hills, IL), filtered through 100-μm nylon mesh (Small Parts, Miami Lakes, FL), washed in RPMI 1640, and counted using a Vicell Counter (Coulter, Hialeah, FL). LN cells were reconstituted and diluted in USP (US pharma) saline (Nurse Assist, Inc) and were infused into CByB6F1 recipients at 5 × 10^6^ LN cells/recipient through lateral tail vein injection. All recipients received 5 Gy total body irradiation (TBI) from a Gammacell 40 (MDS Nordion, Ontario, Canada) source at approximately 0.6 Gy/min four to six hours before LN cell infusion. Recipients were bled and euthanized at day 14 for analyses. In another model, LN cells were obtained from FVB donors and were infused into B6, IL-6^-/-^, TLR2^-/-^ and TLR4^-/-^ recipients at 5 × 10^6^ LN cells/recipient through lateral tail vein injection. All recipients received 6.5 Gy TBI four to six hours before LN cell infusion. Recipients were bled and euthanized at day 9 for analysis.

### Blood and BM cell counting, staining, and flow cytometry

Blood was collected from the retro-orbital sinus into EDTA-coated eppendorf tubes. Complete blood count (CBC) was performed by a HemaVet 950 analyzer (Drew Scientific, Inc., Waterbury, CT). After euthanasia by CO_2_, BM cells were extracted from bilateral tibiae and femurs, filtered through 95 μM nylon mesh, and counted by a Vi-Cell counter (Beckman Coulter, Miami, FL). BM cells were stained with antibody mixtures on ice for 30 minutes in FBS-supplemented RPMI 1640 (Life Technilogies), and acquired using BD FACSCanto II and BD LSRFortessa flow cytometers operated by FACSDiva software (Becton Dickson, San Diego, CA).

Monoclonal antibodies for murine CD3 (clone 145-2C11), CD4 (clone GK 1.5), CD8 (clone 53–6.72), CD44 (clone IM7), CD48 (clone HM48-1), CD62L (clone MEL-14), CD117 (c-Kit, clone 2B8), CD150 (SLAM, clone TC15-12F12.2), erythroid cells (clone Ter119), granulocytes (Gr1/Ly6-G, clone RB6-8C5), stem cell antigen-1 (Sca-1, clone E13-161), and IFN-γ (clone XMG1.2) were all from BioLegend (San Diego, CA). Antibodies were conjugated to fluorescein isothiocyanate (FITC), phycoerythrin (PE), PE-cyanin 5 (PE-Cy5), PE-cyanin 7 (PE-Cy7), allophycocyanin (APC) or brilliant violet 421 (BV421).

### Cytokine measurement

Plasma cytokines of BMF mice were measured using Luminex assay (R&D systems, MN) according to manufacturer’s instruction.

### Statistics

Data was analyzed by variance analyses and multiple comparisons using GraphPad Prism statistical software or JMP statistical discovery software, and presented as means with standard errors. Statistical significance was declared at p<.05.

## Results

### LN cells lacking IL-6, TLR2, and TLR4 are functionally competent to induce BMF

**Fig 1:**
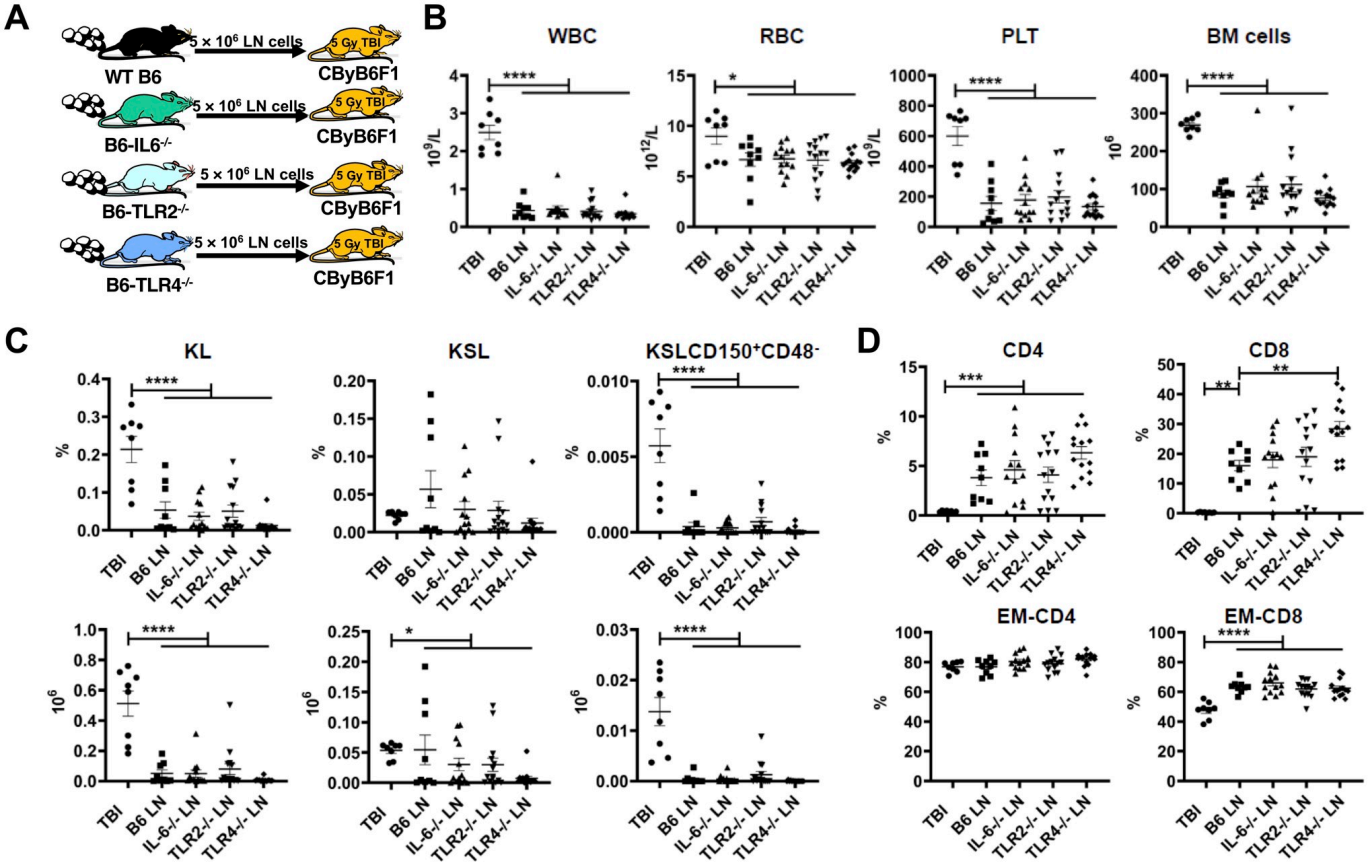
Competence of LN cells from IL-6, TLR2 and TLR4 gene-deletion mice in the induction of immune-mediated BMF. A. Lymph node (LN) cells were extracted from B6, IL-6^-/-^, TLR2^-/-^ and TLR4^-/-^ donors and were infused into gender-matched 5 Gy total body irradiation (TBI 4–6 hours earlier) pre-irradiated CByB6F1 recipients at 5 × 10^6^ LN cells/recipient through lateral tail vein injection. Recipients for B6 (N = 9), IL-6^-/-^ (N = 14), TLR2^-/-^ (N = 14) and TLR4^-/-^ (N = 14) donor LN cells and TBI controls (N = 8) were bled and euthanized 14 days later for analyses. B. WBC, RBCs, platelets, and BM cells in recipient mice that received LN cell infusion from B6, IL-6^-/-^, TLR2^-/-^, and TLR4^-/-^ donors. C. Proportions of CD4^+^ and CD8^+^ T cells, as well as effector memory CD8^+^ (EM-CD8, CD8^+^CD44^+^CD62L^-^) and EM-CD4^+^ (CD4^+^CD44^+^CD62L^-^) T cells in the BM. D. Proportion and total number of KL (Kit^+^Lin^-^), KSL (Kit^+^Sca-1^+^Lin^-^), and KSLCD150^+^CD48^-^ hematopoietic stem and progenitor cells in the four groups that received LN cell infusion. Data shown were means with standard errors. *, P<.05; **, P<.01; ***, P<.001; ****, P<.0001.

**Fig 2:**
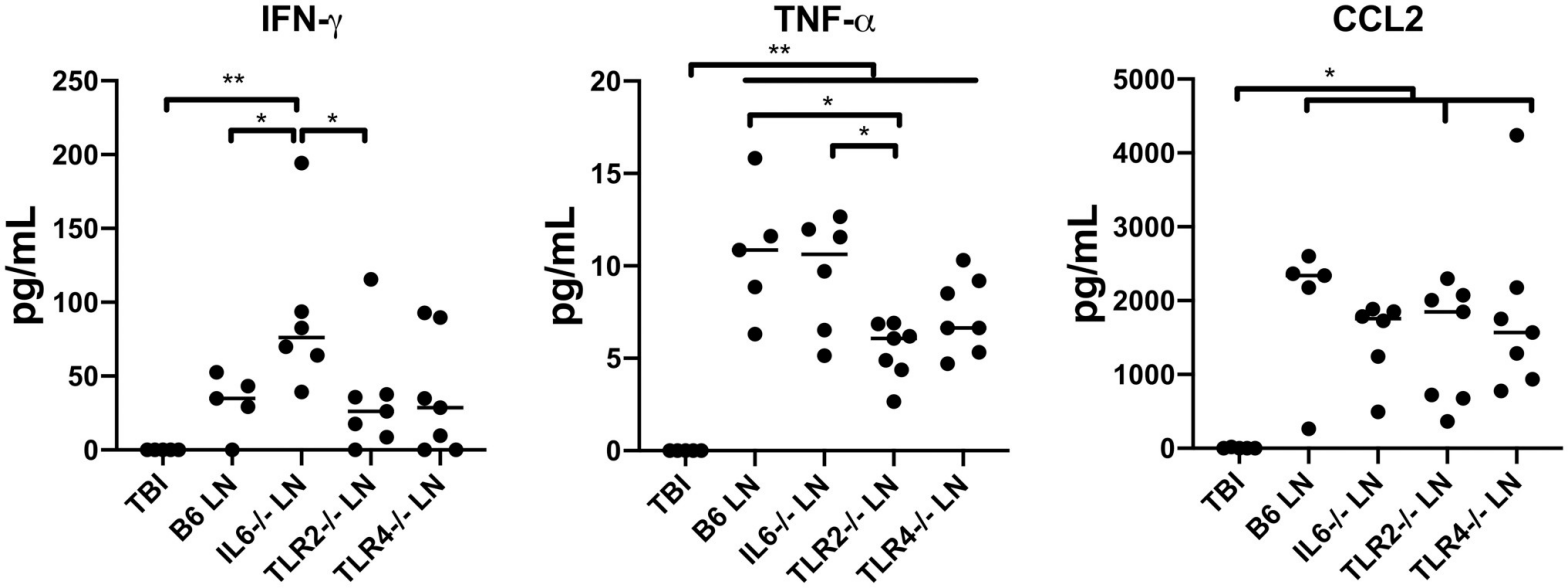
Inflammatory cytokines in BMF mice. IFN-γ, TNF-α, and CCL2 levels in the plasma of CByB6F1 BMF mice induced by infusion of LN cells from B6 (N = 5), IL-6^-/-^ (N = 6), TLR2^-/-^ (N = 7), and TLR4^-/-^ (N = 7) mice. CByB6F1 mice post TBI without LN infusion were controls (TBI, N = 5). *, P<.05; **, P<.01.

In initial experiments, LN cells as a source of effector lymphocytes in our model were obtained from B6, IL-6^-/-^, TLR2^-/-^, and TLR4^-/-^ donors and infused into sub-lethally irradiated CByB6F1 recipients to induce BMF (Fig 1A). Analyses of recipients at day 14 revealed obvious BMF in all recipients showing severe leukopenia, anemia, thrombocytopenia, and BM hypoplasia (Fig 1B). In the BM there were also significant declines in the proportions and total numbers of HSPCs in LN cell-infused recipients relative to TBI controls, as defined by KL (Kit^+^Lin^-^), KSL (Kit^+^Sca-1^+^Lin^-^) and KSLCD150^+^CD48^-^ cell surface markers, but no differences in HSPCs were noted between recipients receiving LN cells from gene deletion donors and those receiving LN cells from normal B6 donors (Fig 1C). Of note, all LN cell-infused animals had significant expansion of CD4^+^ and CD8^+^ T cells, with specific expansion of CD62L^-^CD44^+^ effector memory (EM) CD8^+^ T cells in the BM relative to TBI controls (Fig 1D). When plasma cytokines including IFN-γ, TNF-α, CCL2, Fas Ligand, Granzyme B, IL-1α, IL-1β, IL-2, IL-4, IL-5, IL-10, IL-12 p70, IL-17A, and IL-17E were measured, we found Th1 and inflammatory cytokines such as IFN-γ, TNF-α, and CCL2 in recipient BMF mice were higher than in TBI control CByB6F1 mice. The other cytokines were either undetectable or demonstrated no difference between BMF and control mice. The recipient mice that received IL-6^-/-^ LN infusion had significantly higher IFN-γ in the plasma than did the animals that received LN infusion from wild-type B6 or TLR2^-/-^ donors, while the recipient mice that received TLR2^-/-^ LN infusion had lower levels of TNF-α than those that received LN infusion from wild-type B6, IL-6^-/-^, and TLR4^-/-^ mice (Fig 2).

### Susceptibility of IL-6, TLR2, and TLR4 gene-deletion mice to immune-mediated BMF

**Fig 3:**
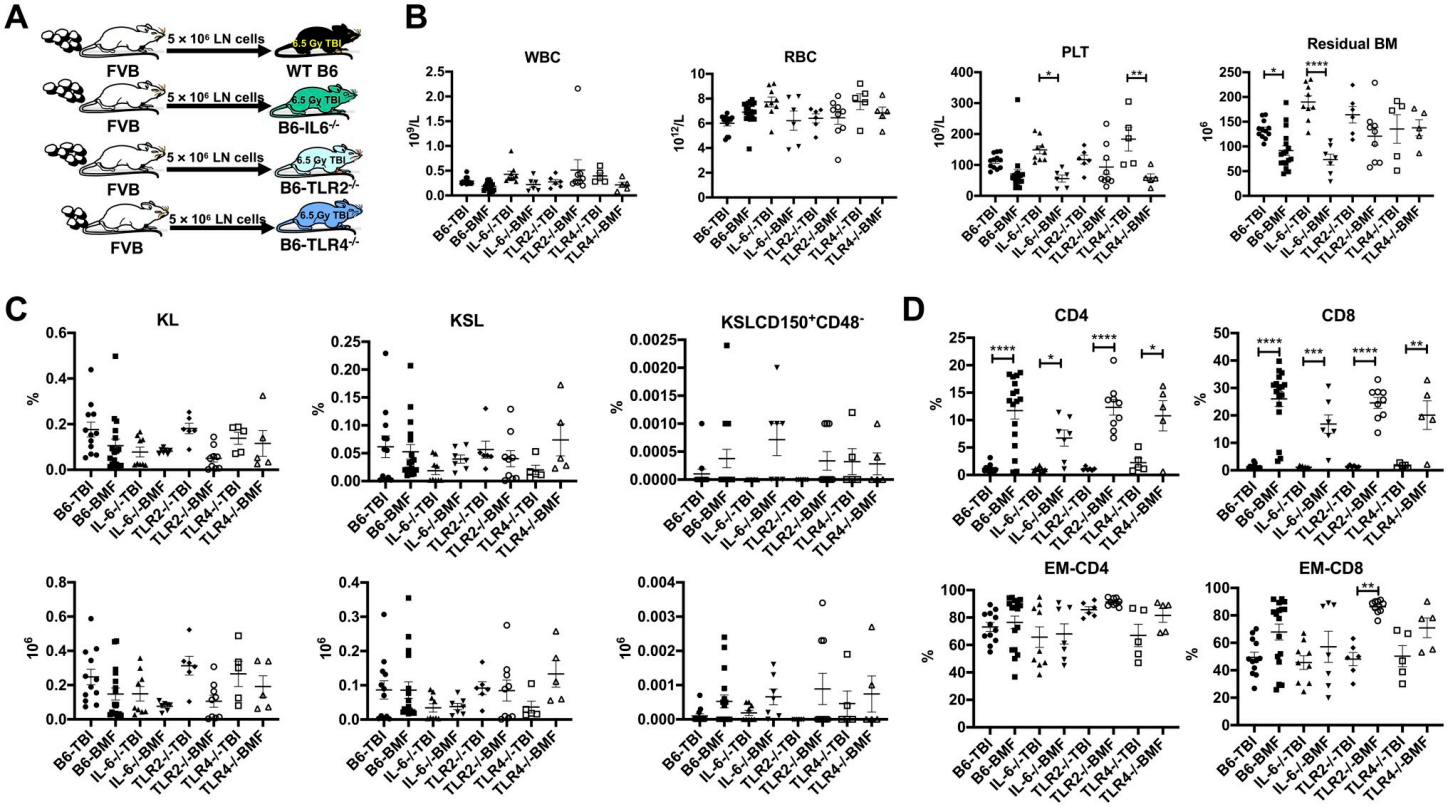
Maintained susceptibility to immune-mediated BMF following germline deletion of IL-6, TLR2 and TLR4. A. B6, IL-6^-/-^, TLR2^-/-^ and TLR4^-/-^ mice were pre-irradiated with 6.5 Gy TBI 4–6 hours early without (TBI controls, N = 12, 9, 6, 5) or with the infusion of at 5 × 10^6^ LN cells/recipient from gender-matched FVB donors (BMF, N = 17, 7, 9, 5). Recipient mice were bled and analyzed at day 9 following LN cell infusion. B. WBC, RBCs, platelets, and residual BM cells (BM cells excluding CD4 and CD8 T cells) in B6, IL-6^-/-^, TLR2^-/-^, and TLR4^-/-^ recipient mice that received LN infusion from FVB donor mice. C. Proportions of BM CD4^+^, CD8^+^, EM-CD4^+^, and EM-CD8^+^ T cells in the BM of BMF-IL-6^-/-^, BMF-B6, BMF-TLR2^-/-^, and BMF-TLR4^-/-^ mice relative to their TBI controls. D. Proportion and total number of KL (Kit^+^Lin^-^), KSL (Kit^+^Sca-1^+^Lin^-^), and KSLCD150^+^CD48^-^ hematopoietic stem and progenitor cells in the BM of B6, IL-6^-/-^, TLR2^-/-^, and TLR4^-/-^ recipient mice that received LN infusion from FVB donor mice. *, P<.05; **, P<.01; ****, P<.0001.

In a reverse model, we used wild-type B6, IL-6^-/-^, TLR2^-/-^, and TLR4^-/-^ mice as recipients of MHC mismatched FVB LN cells (Fig 2A). Marrow failure develops more rapidly in this model than when using CByB6F1 as recipients (H2^b/b^ to H2^b/d^), likely due to the greater MHC mismatch between donors and recipients (H2^q/q^ to H2^b/b^). Two out of 9 IL-6^-/-^ recipients died at 7 and 8 days due to rapid disease progression therefore we analyzed recipient mice at 9 days following LN infusion. There were reductions in platelets in the peripheral blood in all LN cell-infused recipients relative to their respective TBI controls, while a decrease in WBC was noted only in wild-type B6 and IL6^-/-^ recipients (Fig 3B). Day 9 post LN infusion is a stage of large T cell expansion, which dominates BM cellularity; therefore, we used residual BM cell counts excluding the expanded CD4^+^ and CD8^+^ T cells to reflect BM damage. There were significant decreases in total residual BM cells had significant decreases in wild-type B6 and IL-6^-/-^ recipients compared to their respective TBI controls. The decline of residual BM cells was not prominent in TLR2^-/-^ and TLR4^-/-^ recipients, suggesting milder BM damage than wild-type and IL-6^-/-^ animals (Fig 3B). IL-6^-/-^ recipients appeared to suffer more severe BM damage as indicated by earlier mortality and fewer residual BM cell numbers relative to other groups of recipients (Fig 3B). The proportion and total number of BM KL was reduced in all groups of recipient mice post LN-infusion; the frequency of BM KSL cells decreased significantly in B6 mice, but the difference did not reach significance in IL-6^-/-^, TLR2^-/-^ and TLR4^-/-^ mice relative to controls (Fig 3C). All LN cell-infused recipients showed large increases in the proportions of CD4^+^ and CD8^+^ T cells in the BM relative to TBI controls, with significant increases in EM-CD8^+^ cells, except in TLR4^-/-^ mice, while EM-CD4^+^ was increased only in B6 BMF mice relative to TBI controls (Fig 3D). Despite variations in some measurements, the expansion and activation of CD4^+^ and CD8^+^ T cells were drastic in all recipients following FVB LN cell infusion.

**Fig 4:**
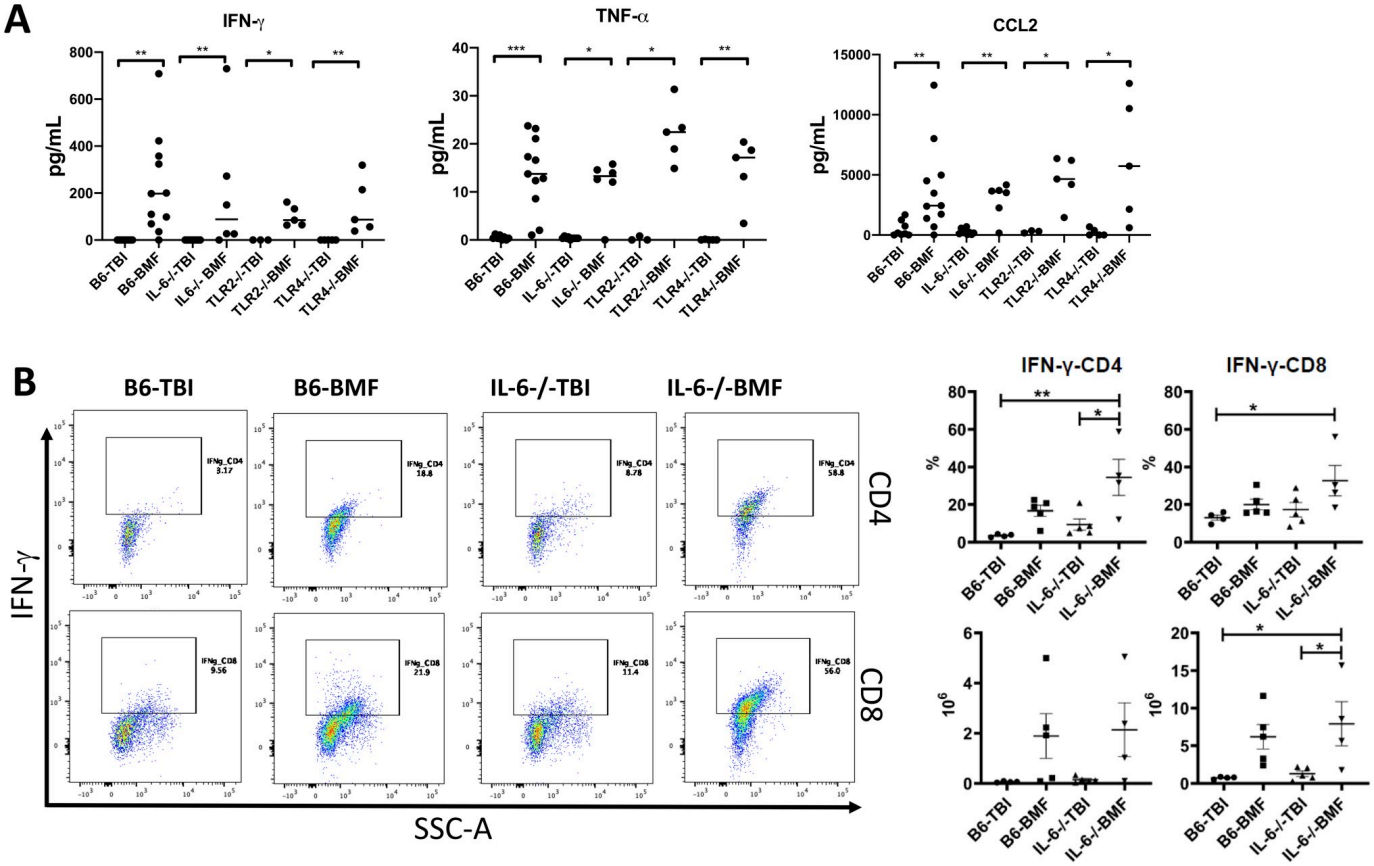
Inflammatory cytokines in B6, IL-6^-/-^, TLR2^-/-^, and TLR4^-/-^ recipient mice following FVB LN infusion. A. IFN-γ, TNF-α, and CCL2 levels in the plasma of B6 (N = 11), IL-6^-/-^ (N = 6), TLR2^-/-^ (N = 5), and TLR4^-/-^ (N = 5) recipient mice following FVB donor LN infusion as well as cytokines in their corresponding TBI control mice (N = 8, 9, 3, 5). The cytokines were measured by Luminex assay. B. Intracellular IFN-γ levels in CD4^+^ and CD8^+^ T cells in the BM of B6 (N = 5) and IL-6^-/-^ (N = 4) recipient mice as well as their corresponding TBI control mice (N = 4, 5) detected by flow cytometry. *, P<.05; **, P<.01; ***, P<.001.

Plasma levels of Th1 and inflammatory cytokines such as IFN-γ, TNF-α, and CCL2 in all recipient BMF mice were higher than in respective TBI controls (Fig 4A). Given greater severity of disease in IL6^-/-^ recipients, we speculated that T cells which infiltrated the BM of IL-6^-/-^ mice had higher intracellular levels of IFN-γ, the most important Th1 cytokine in the pathogenesis of murine AA. By flow cytometry, we observed that IL-6^-/-^ recipients had more prevalent intracellular IFN-γ CD4^+^ and CD8^+^ T cells in the BM than did wild-type B6 recipient mice, but the number of IFN-γ-secreting T cells was similar between the two BMF groups (Fig 4B).

Thus, recipient mice carrying germline deletion of IL-6, TLR2, and TLR4 were all susceptible to FVB LN cell-induced BM damage, but the disease appeared more severe in IL-6 deficient mice.

## Discussion

Using well characterized murine models, we aimed to gain some insight into the roles of components of the innate immune system in the development of immune AA. We demonstrate that T cells lacking TLR2, TLR4, or IL-6 successfully induced severe pancytopenia and BMF. Likewise, recipient mice with TLR2, TLR4, or IL-6 gene deletions were all susceptible to a variable degree of immune-mediated BMF.

TLRs have been sporadically investigated in different BMF pathologies, but not in relation to AA specifically. For example, innate immune system activation was studied in a zebrafish model of Diamond-Blackfan anemia, a constitutional anemia due to mutations that cause deficiency of ribosomal proteins (RP). The study demonstrated that RP-deficient zebrafish treated with a TLR3 inhibitor decreased IFN activation, acute phase responses, and apoptosis, and improved hematopoiesis [35]. In the myelodysplastic syndromes (MDS), inhibition of TLR2 in cultured BM CD34^+^ cells from patients with lower risk MDS led to increased formation of erythroid colonies [36]. In del (5q) MDS, murine studies have suggested secretion of DAMPs, S100A8 and S100A9, which are endogenous activators of TLR4 [37, 38], and linked to hematopoietic dysplasia and pathologic erythroid differentiation [37, 39].

Other studies have focused on the effect of TLR stimulation on hematopoiesis. HSPCs express TLRs and acute TLR stimulation, via the MyD88 pathway, enhances production of innate immune cells, in differentiation of myeloid progenitors [19, 40–44], and lymphoid progenitors to become dendritic cells [19, 42, 45]. A murine study done injecting low dose zymosan, a TLR2 ligand, into the peritoneal cavity of mice resulted in increased number of innate lymphoid cells [45]. These published studies demonstrate that short term or single exposure to TLR agonists increases HSPC activity. On the other hand, long term exposure to TLR agonists affects HSPCs negatively: When mice were exposed to small daily doses (6 μg) of LPS, a TLR4 ligand, over a 4–6 week treatment period (to mimic chronic inflammation), long-term HSPCs lost the ability to sustain lymphoid differentiation on serial transplantation [37, 43]; Another study demonstrated daily doses of 1 μg of LPS over a month caused dramatic reduction in long-term, but not short term HSPC repopulating activity, and no shift in the balance between myeloid and lymphoid differentiation [37, 44]; Some evidence also suggests that chronic or aberrant TLR stimulation may result in MDS and be important in other hematologic malignancies [19]; Chronic innate immune stimulation of KDM6B, an epigenetic regulator that mediates transcriptional activation during differentiation, via LPS resulted in leukopenia, dysplasia, and compromised repopulating function of BM HSPCs [19, 46]. These findings suggest that the specific effects of TLR agonists on HSPCs are dependent on dose or duration of exposure.

IL-6 also has a significant effect on hematopoiesis, especially for marrow suppression in the setting of acute myeloid leukemia (AML). While the common explanation of marrow suppression in leukemia has been overcrowding by blasts, many patients with incomplete BM involvement and low leukemic burden experience clinical symptoms of BMF. AML blasts produce inflammatory cytokines, including IL-6, thereby inducing progenitor depletion, endosteal endothelial remodeling, and decreasing colony forming potential of normal CD34^+^ cells [47–49]. Differential up-regulation and secretion of IL-6 by AML blasts occurs in vitro in patient samples, and in vivo in murine xenograft models. Paracrine effects of IL-6 suppressed erythroid differentiation and caused anemia in animals; a neutralizing IL-6 antibody reversed this effect in vitro and in vivo [50]. As with TLRs, acute versus chronic stimulation by IL-6 can have opposing effects on hematopoiesis. In adults, IL-6 released from HSPCs in response to acute stressors such as sepsis, chemotherapy, or in a post-transplant setting, can also promote myelopoiesis [37, 51] In the setting of prolonged inflammation, chronically elevated IL-6 levels cause direct mitochondrial damage in developing erythroid cells, resulting in impaired hemoglobin production [37, 52].

Our laboratory has examined the changes of IL-6 in human and murine AA [14]. Th17 cells, a subset of CD4^+^ T cells, function as immune effectors in the setting of inflammation, infection, and autoimmunity. In mice, the differentiation of Th17 cells from naive CD4^+^ T cells requires IL-6 and TGF-β; in humans, IL-6 is also involved in Th17 differentiation. We isolated Th17 cells from patients’ blood and BM and found that the total number of CD3^+^CD4^+^IL-17^+^ T cells was increased in patients with AA at presentation compared with healthy controls. Further, in the murine model, early treatment with anti-IL-17 antibody attenuated the severity of BMF; recipients treated with anti-IL-17 monoclonal antibody had fewer Th1 cells and more Treg cells along with an insignificant decline in plasma IL-6 level at this time point [14].

In the current work, inflammatory cytokines in the recipient mice were measured in both experimental models. Using gene deletion mice as donors, plasma IFN-γ was found at higher levels in the CByB6F1 BMF recipient mice from IL-6^-/-^ donors compared to the CByB6F1 BMF recipient mice from wild-type B6 and TLR2^-/-^ donors (Fig 2), which correlates with the results from the second model where IFN-γ was found at higher level in both CD4^+^ and CD8^+^ T cells of IL-6^-/-^ recipient mice compared to wild-type B6 recipient animals (Fig 4B), suggesting IL-6 deficiency in either donor T cells or host environment promoted IFN-γ production. This antagonistic result between IL-6 and IFN-γ in our animal models is intriguing given IL-6 and IFN-γ tend to be synchronous in settings of inflammation [53]. Further, IFN-γ was reported to enhance IL-6 production in LPS-stimulated monocytes [54]. We speculate that the greater production of T cell-derived IFN-γ in IL-6^-/-^ BMF mice relative to wild-type B6 BMF mice in our study might reflect a compensatory effect of IFN-γ on IL-6.

In contrast to the data from the literature mentioned, and their interpretation, in our current work we could not detect a significant role for innate immunity in experimental immune AA. Of course, the pathways involved in hematopoiesis in different settings of inflammation are complex and numerous. Furthermore, the murine models of immune AA are based on responses to known and strong antigenic stimuli, major H2 antigens, or minor histocompatibility antigens, whereas the provocative antigens in human immune AA are largely unidentified. Our study looked at only two of the ten human TLRs and IL-6 in single gene knockout models. We cannot exclude the possibility that depletion of one TLR gene might be compensated by other TLR genes. Double-gene knockout or multiple gene knockout TLR work might be necessary to understand the roles of TLRs in BMF. The results of our study are similar to a study we recently reported on IL-18 [17], which is also known as IFN-γ inducing factor and another component of the innate immune system’s cascade of activation of the cytotoxic T cell response. IL-18 levels were elevated in the sera of severe AA patients and BMF mice. However, deletion of the IL-18 gene in donor LN cells or deletions of either IL-18 or IL-18 receptor in recipient animals did not alleviate induction of BMF. Thus, in the model, IL-18 appeared to be dispensable [17]. Distinct from the apparent dispensable roles of IL-18, as well as of TLRs and IL-6 in current study, we have systematically confirmed the determinate roles of IFN-γ and TNF-α in experimental BMF using gene knockout mice [11, 12]. In one study, LN cells from donor mice carrying germline deletion of IFN-γ were incapable of inducing severe BMF relative to LN cells from wild-type B6 donor mice [11]. In another study LN cells from TNF-α receptor (TNF-rsf1a^-/-^1b^-/-^) donors were also incapable of inducing severe BMF while LN cells from TNF-α^-/-^ donors were fully functional in causing severe marrow damage [12].

The results of the current study suggest that TLR2, TLR4, or IL-6, individually do not play an essential role in the induction of BMF. Further research could examine other components of the innate and adaptive immune system for better understanding of the molecular pathophysiology contributing to AA. The absence of a marked influence of innate immune roles in this model of human disease does emphasize the importance of the more specific adaptive response in the mechanisms in these models.

## Supporting information

S1 File IIBMF-03-04-12-13-updated. (PZFX)
